# Fifteen years of tuberculosis and HIV diagnostic services in Brazil: disruption, regional disparities, and recovery before, during, and after the COVID-19 pandemic

**DOI:** 10.1016/j.ijregi.2025.100796

**Published:** 2025-10-31

**Authors:** Luanne Karolyne Leal dos Santos, Yan Mathias Alves, Reginaldo Bazon Vaz Tavares, Marcela Antunes Paschoal Popolin, Nathalia Zini, Ione Carvalho Pinto, Pedro Fredemir Palha, Aline Aparecida Monroe, Erica Chimara, Ricardo Alexandre Arcêncio

**Affiliations:** 1 Department of Maternal-Infant and Public Health Nursing, Ribeirão Preto College of Nursing, University of São Paulo, Ribeirão Preto, Brazil; 2 Federal University of Tocantins, Palmas Campus, Tocantins, Brazil; 3 Laboratory of Tuberculosis and Mycobacterioses, Adolfo Lutz Institute, São Paulo, Brazil; 4 Brazilian Tuberculosis Research Network, REDE-TB, São Paulo, Brazil

**Keywords:** Diagnostic testing, Health care disparities, Tuberculosis, HIV, COVID-19, Brazil

## Abstract

**Objectives:**

This study analyzes fifteen years of nationwide trends and regional disparities in tuberculosis (TB) and HIV diagnostics in Brazil, examining the impacts of the COVID-19 pandemic and the uneven recovery of diagnostic services across different regions.

**Methods:**

This nationwide ecological study analyzed monthly data on TB and HIV diagnostic tests performed in Brazil from 2010 to 2024, utilizing real-world data. Temporal trends and interrupted time-series analyses evaluated the immediate and progressive effects of the COVID-19 pandemic. Spatial patterns and autocorrelation were explored using bivariate Moran’s I and Kernel density estimation.

**Results:**

Results showed significant abrupt declines during the COVID-19 pandemic for smear microscopy (–16.4%), culture (–21.4%), and HIV (–16.2%). Post-pandemic, Xpert MTB/RIF showed the highest monthly increase (+3.8%; 95% confidence interval: 2.9-4.7), while smear microscopy declined (–1.2%; 95% confidence interval: –2.0 to –0.5). Spatial analysis revealed pronounced regional heterogeneity, with Northern and Northeastern municipalities facing the greatest disruptions. The Xpert MTB/RIF test demonstrated greater resilience, with evident growth in its distribution and implementation over time. However, this increase resulted in a decrease in the conduction of smear microscopy tests and highlighted territorial disparities, since only 167 municipalities (3%) have Xpert MTB/RIF assays, mainly in the Southeast.

**Conclusions:**

COVID-19 significantly disrupted traditional TB and HIV diagnostics in Brazil, while there was an evident increase and resilience of Xpert MTB/RIF, reducing the use of smear microscopy tests. Policy efforts should prioritize the equitable expansion of molecular diagnostic technologies across all regions. This approach will improve early TB detection and drug resistance testing, thereby reducing diagnostic disparities and strengthening health system resilience toward achieving the End TB Strategy.

## Highlights

• The COVID-19 pandemic caused disruptions in tuberculosis and HIV diagnostic testing
• The post-pandemic period was marked by a gradual recovery in tuberculosis and HIV diagnosis
• Spatial analyses reveal clustered gains where lab capacity was preexisting
• The expansion of Xpert MTB/RIF resulted in a decrease in the use of smear microscopy
• Brazilian regions are still affected by inequality in the spatial distribution of diagnostic tests

## Introduction

Tuberculosis (TB) remains a significant public health issue and continues to be an important cause of global mortality, with approximately 10.8 million new cases diagnosed in 2023, the highest number since global monitoring of the disease began in 1995 [1].

In the Brazilian context, diagnostic methods for TB include clinical, bacteriological, imaging, histopathological, and other approaches [2]. Among the most commonly used are sputum smear microscopy [3]; culture for mycobacteria, considered the gold standard for disease identification [4]; drug susceptibility testing (DST), which checks for drug resistance [2]; and the rapid molecular test [5].

Although there are recommendations by the World Health Organization (WHO) for other molecular tests in TB diagnosis, such as rapid molecular tests based on real-time polymerase chain reaction (PCR) [6], Brazil has officially approved the use of Xpert MTB/RIF, introduced in 2014 and replaced by the new version, Xpert MTB/RIF Ultra, in 2019 [7]. In addition to this test, other molecular tests are under evaluation and incorporation processes with the National Committee for Health Technology Incorporation (Conitec) and the Brazilian Health Regulatory Agency (Anvisa), aiming to further expand the molecular diagnostic options available in the country [8,9].

For performing the Xpert MTB/RIF and Xpert MTB/RIF Ultra, the GeneXpert^Ⓡ^ system is used, approved by the WHO in 2010 [10]. The test is based on PCR methodology, which performs real-time amplification and detection of *Mycobacterium tuberculosis* DNA fragments while simultaneously checking for rifampicin resistance by gene mutations [11]. It is considered cost-effective due to its speed, efficacy in delivering results, and lack of requirement for specific sample preparation or highly specialized human resources [7].

Given these advantages, the WHO recommends molecular tests as the preferred initial method for TB diagnosis. However, bacteriological culture remains essential, especially for confirming the final diagnosis, investigating additional drug resistances, and monitoring treatment. This is because culture provides detailed information that molecular tests cannot capture [12].

In Brazil, the expansion and decentralization of the GeneXpert^Ⓡ^ system have been a priority to enhance TB diagnosis through molecular testing. However, this process faces challenges due to structural issues and territorial inequalities in the distribution of resources [6]. The last public record of equipment expansion was in 2018, increasing from 178 to 248 machines, covering only 128 municipalities and about 65% of annual notifications of new cases [13]. After the onset of the COVID-19 pandemic, no public records regarding the expansion of the Xpert MTB/RIF network were identified, highlighting gaps in coverage and post-intervention monitoring.

Regarding HIV, the WHO recommends universal testing for TB-HIV coinfection using serological tests for HIV [14]. In 2023, the global coverage for TB-HIV testing remained at 80%, while in Brazil it reached 84%, with 11.4% of patients identified as coinfected [1], [15].

Over the past decade, and more than 35 years after the creation of Brazil’s Unified Health System (SUS), access to TB and HIV testing has expanded nationwide [16]. However, no single study has examined these trends at the national level, as most research has been limited to specific regions or short time periods.

A comprehensive study combining long-term temporal and spatial analyses is needed to understand how service disruptions and recoveries have varied across the country and regions. Identifying inequalities is crucial to guide targeted policies, as health crises like COVID-19 worsen existing disparities. Strengthening diagnostic access and resilience in vulnerable areas is key to improving health outcomes and equity during and after emergencies, thereby reinforcing the agenda to end the TB Strategy, aiming to reduce TB mortality by 95% and the incidence of new cases by 90% in the country by 2035 [17].

Therefore, this study aims to fill the gap by analyzing nationwide temporal trends of TB and HIV diagnostic testing from 2010 to 2024 and to assess the immediate and longer-term impacts of the COVID-19 pandemic on diagnostic services, including spatial heterogeneities and service recovery across different regions.

## Methods

### Study location and design

This is an ecological study involving spatial analysis and time series, utilizing real-world data.

The study covers the entire Brazilian territory and its five macro-regions (North, Northeast, Southeast, South, and Central-West). Brazil has an area of 8,510,000 km² and an estimated population of 211.1 million inhabitants distributed across 5,570 municipalities [18]. In 2024, the TB incidence rate in Brazil was 39.7 cases per 100,000 inhabitants, with notable regional variations. The North (62.7/100,000) and Southeast (43.4/100,000) regions showed the highest incidences, followed by the Northeast (36.2/100,000) and South (30.0/100,000). The Central-West region had the lowest incidence, with 24.3 cases per 100,000 inhabitants [15].

### Population

The study included individuals who underwent diagnostic testing for TB, registered in Brazil's Information System for Notifiable Diseases (SINAN). The time interval analyzed in this study covers the period between January 1, 2010, and December 31, 2024, and all notifications were extracted from SINAN in June 2025.

### Variables and definitions

*Dependent variables (outcomes)*

(a) Monthly number of TB diagnostic tests performed (Xpert MTB/RIF; sputum smear microscopy; mycobacterial culture; DST; rapid test for HIV [TB-HIV coinfection]).

*Definition: Absolute number of tests performed for TB diagnosis, categorized by type of test, each month from 2010 to 2024.*

In this article, the term Xpert MTB/RIF will be used generically to refer to both versions of the rapid molecular test (Xpert and Ultra), since there was no temporal or spatial distinction regarding the use of each. The DST refers specifically to the phenotypic susceptibility testing performed using the MGIT (Mycobacteria Growth Indicator Tube) system.

(b) Annual proportions of tests performed

*Definition: The number of tests performed by year, divided by the total number of all types of tests, multiplied by 100.*

(c) Monthly number of TB cases

*Definition: Total number of TB cases by municipality, from 2010 to 2024.*

### Independent variables

Rationale for time period classification:

The study period was divided into pre-pandemic, pandemic intervention, and post-pandemic phases regarding the performance of HIV and TB diagnostic testing.

(i) Pre-pandemic: 2010-2019. The period represents baseline trends in testing, reflecting routine diagnostic activities and programmatic stability for both HIV and TB. The term "pre-pandemic" refers to a concept used to define the epidemiological baseline, allowing historical trends to be compared and abrupt changes triggered by health crises to be evaluated.
(ii) Intervention period: 2020-2021 (onset of the pandemic). Corresponds to the height of COVID-19-related disruptions, when social distancing, healthcare service restructuring, and resource reallocation affected access to and availability of diagnostic tests, including smear microscopy, culture, Xpert MTB/RIF, and rapid HIV tests.
(iii) Post-intervention: 2022-2024. Captures the gradual restoration of health services, resumption of testing activities, and policy adjustments aiming to recover routine surveillance and control of co-infections. This temporal classification respects the natural history of both infections and aligns with the study’s objective to assess COVID-19’s indirect effects on essential diagnostic services.

### Data analysis

#### Temporal analysis

For this stage, monthly time series were constructed for the number of tests performed, stratified by type of test (smear microscopy, culture, Xpert MTB/RIF, DST, and rapid HIV test), for Brazil and its macro-regions, from 2010 to 2024.

To analyze monthly trends in diagnostic test counts while accounting for autocorrelation, we applied Prais-Winsten regression and interrupted time series models based on the literature [19,20].

#### Prais-Winsten model

This model corrects for first-order autocorrelation [AR(1)] in residuals, improving estimation efficiency [21]. The basic log-linear regression is:

log(Yt)=β0+β1t+ϵt

where Y_t_ is the number of tests at month t, β_0_ is the intercept, β_1_ is the slope representing the linear monthly trend, and ϵ_t_ is the error term with AR(1) structure. The monthly percent change (MPC) is calculated as:

MPC=(10β1−1)x100

interpreted as the average percent increase or decrease in tests per month. The interpretation of the MPC: positive values indicate an increasing trend, and negative values indicate a decreasing trend [19]. The final trend classification was defined as “increasing” when β >0 and *P* <0.05, “decreasing” when β <0 and *P* <0.05, and “stationary” when *P* ≥0.05, with results presented separately by test type, country, and macro-region. The statistical significance of this coefficient was assessed using the point estimate, standard error, and confidence interval [19].

### Interrupted time-series model

To assess the impact of the COVID-19 onset as an intervention point, we extended the Prais-Winsten model [20] to:

log(Yt)=β0+β1t+β2Dt+β3Pt+ϵt

where $D_{t}$ is an indicator variable (0 before intervention, 1 after) capturing the immediate level change, and $P_{t}$ counts months post-intervention, capturing trend changes. This allows separation of pre- and post-intervention trends and level shifts. The MPC is calculated similarly for each segment, illustrating the pandemic's disruption and recovery effects [20]. This specification ensures robust estimation of monthly trends and intervention effects, accounting for autocorrelation and seasonality.

Furthermore, annual time-series analyses were applied to determine the percentage use of TB diagnostic tests (smear microscopy, culture, Xpert MTB/RIF, DST), enabling a comparison to identify the percentage utilization of each test.

### Spatial analysis

For this stage, all 5570 Brazilian municipalities were considered as spatial units of analysis. Spatial analyses were conducted using TB diagnostic tests (smear microscopy, culture, Xpert MTB/RIF, DST), considering the pre-COVID-19 period (2010 to 2019) as the pre-intervention period and the post-COVID-19 period (2020 to 2024) as the post-intervention period. To create the maps, municipal and regional shapefiles of Brazil provided by the Brazilian Institute of Geography and Statistics (IBGE) were used [22]. In this phase, the number of diagnostic tests performed was stratified by municipality and by Brazil’s five macro-regions (North, Northeast, Southeast, South, and Central-West). For this analysis, the queen contiguity was considered as the spatial weights matrix.

### Global spatial autocorrelation

To estimate global spatial autocorrelation, the Moran’s I statistic was employed [23], which measures the correlation between the values of a variable $x_{i}$ and its spatial lag. For each unit $i,\;z_{i}=x_{i}-\bar{x}$ is defined, and the spatial lag is given by $\left(W_{Z}\right)i=\sum_{j}w_{ij}z_{j}$, where $w_{ij}$ are the elements of the spatial weights matrix. Moran’s I is calculated as:

$$I=\frac{\sum_{i}\sum_{j}z_{i}z_{j}*w_{ij}/S_{0}}{\sum_{i}z_{i}^{2}/n}$$

where n is the number of spatial units and $\bar{x}$ is the mean of x. A positive Moran’s I indicates clustering of similar values, while a negative value indicates spatial dissimilarity [23].

### Interpretation of Bivariate Local Moran's I Clusters

To identify spatial patterns between two variables, the Bivariate Local Moran’s I was used [23]. This method assesses the relationship between a variable at a given location and the average of the neighboring values of another variable, allowing the identification of whether areas with high values tend to be located near areas with high or low values of the other analyzed variable. The Bivariate Local Moran’s I is expressed as:

$$I_{i}^{B}=x_{i}\sum_{j}w_{ij}y_{j}$$

where $x_{i}$ represents the standardized value of the variable X at location $i,\;y_{i}$ corresponds to the standardized value of the variable Y at the neighboring locations j, and $w_{ij}$ are the elements of the spatial weights matrix that define the neighborhood relationships [23].

To implement this analysis, a point shapefile was constructed in which each municipality in Brazil was represented as a point on the map. The weight assigned to each point corresponded to the number of diagnostic tests performed, enabling the visualization of spatial patterns and the identification of areas with higher or lower intensity of test usage [23]. For the spatial analyses of the global and local bivariate Moran’s I, the GeoDa software (version 1.22) was used.

### The kernel density estimator

The kernel density estimator (KDE) was employed to identify areas with higher concentrations of TB diagnostic test usage. KDE is a non-parametric smoothing technique that generates a continuous intensity surface from discrete point events, facilitating visual detection of spatial clustering and hotspot identification where tests are more frequently performed. The method involves defining a search radius (bandwidth) around each point and estimating the density of neighboring events within this radius, producing a smooth surface that highlights areas of increased or decreased concentration [24].

The KDE at location x is estimated by:

$$f\left(x\right)=\;\frac{1}{nh}\sum_{i=1}^{n}K\left(\frac{x-X_{i}}{h}\right)$$

where n is the number of observed points, h is the bandwidth controlling the smoothness of the density estimate, $x_{i}$ are the locations of observed events, and K(·) is the kernel function, typically a symmetric function like the Gaussian or quartic kernel that decreases with distance from each point. The bandwidth h determines how smoothly the resulting surface reflects the underlying event pattern, balancing detail and noise [24].

To analyze the spatial distribution of Xpert MTB/RIF machine availability, we constructed a map by georeferencing all municipalities with access to this technology. Additionally, a kernel density analysis [24] was applied to identify areas with greater access. For the final visualization and map design, QGIS software (version 3.40.6) was used.

### The generalized least squares

The generalized least squares (GLS) model was employed to estimate the relationship between TB diagnostic test use and the number of reported cases while explicitly addressing heteroscedasticity and autocorrelation detected in residuals from ordinary linear regression. GLS allowed modeling a variance-covariance structure of residuals, which accommodates non-constant variance (heteroscedasticity) and autocorrelation simultaneously [25]. Two separate GLS models were fit (pre- and post-pandemic) with TB case counts as the dependent variable and diagnostic tests as independent variables. Model fit assessment involved the Akaike Information Criterion and statistical tests like the Breusch-Pagan test for heteroscedasticity. This analysis was performed in R software (version 4.3.1).

## Results

### Overview of diagnostic tests (2010-2024)

Between 2010 and 2024, a total of 3,078,101 TB diagnostic tests were performed nationwide. Among these, sputum smear microscopy accounted for 31.4% (n = 965,644), mycobacterial culture 14.9% (n = 459,596), Xpert MTB/RIF 12.0% (n = 368,724), DST 5.4% (n = 167,174), and rapid HIV tests 36.3% (n = 1,116,963).

The Brazilian macro-region with the highest number of tests was the Southeast (n = 1,503,726; 48.8%), followed by the Northeast (n = 690,356; 22.4%), South (n = 404,577; 13.2%), North (n = 335,858; 10.9%), and Central-West (n = 143,584; 4.7%).

### Temporal trends of diagnostic tests

**Figure 1:**
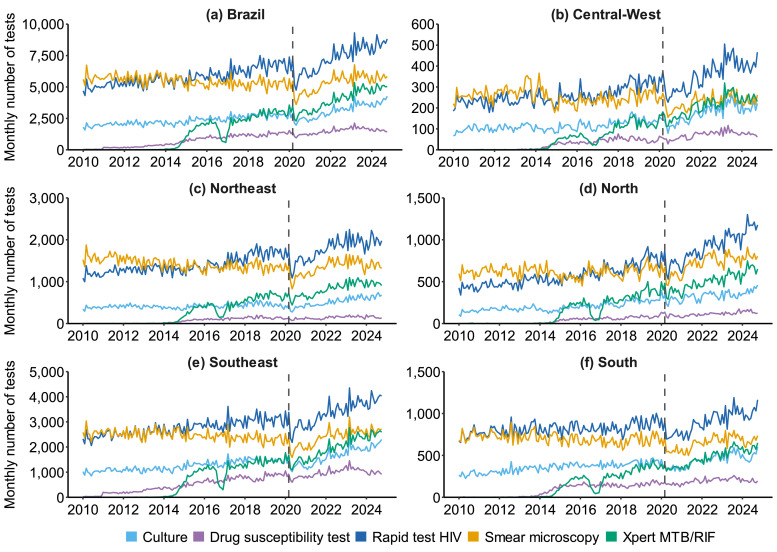
Monthly time series of tuberculosis diagnostic tests performed nationally and by macro-regions (2010–2024), considering the COVID-19 intervention point. (a) Number of tests performed in Brazil (yellow = smear microscopy; blue = culture; dark blue = rapid test HIV; purple = drug susceptibility test; green = Xpert MTB/RIF). (b) Number of tests performed in the Central-West region (yellow = smear microscopy; blue = culture; dark blue = rapid test HIV; purple = drug susceptibility test; green = Xpert MTB/RIF). (c) Number of tests performed in the Northeast region (yellow = smear microscopy; blue = culture; dark blue = rapid test HIV; purple = drug susceptibility test; green = Xpert MTB/RIF). (d) Number of tests performed in the North region (yellow = smear microscopy; blue = culture; dark blue = rapid test HIV; purple = drug susceptibility test; green = Xpert MTB/RIF). (e) Number of tests performed in the Southeast region (yellow = smear microscopy; blue = culture; dark blue = rapid test HIV; purple = drug susceptibility test; green = Xpert MTB/RIF). (f) Number of tests performed in the South region (yellow = smear microscopy; blue = culture; dark blue = rapid test HIV; purple = drug susceptibility test; green = Xpert MTB/RIF).

Over the years, rapid HIV tests and smear microscopy showed the highest monthly values (Figure 1). Compared to other tests, DST appeared in the Southeast in 2010 and was present in other regions starting in 2014. Xpert MTB/RIF was implemented beginning in 2014, showing a sharp drop at the end of 2016 and the beginning of 2017. Another pronounced drop across all tests was observed at the beginning of 2020, between March and April, coinciding with the onset of the COVID-19 pandemic.

During the pre-pandemic period (2010–2019), culture, rapid HIV testing, and Xpert MTB/RIF showed statistically significant increases across Brazil, with MPC for Xpert MTB/RIF ranging from 3.5% to 4.8% (95% CI = 1.2-7.6, *P* <0.05) (Supplementary Table 1). Conversely, smear microscopy generally declined except for a modest increase in the North region (MPC = 0.11%, 95% CI = 0.05-0.18, *P* = 0.01).

Based on the results shown in Table 1, an abrupt decline was observed in smear microscopy, culture, and HIV tests at the beginning of the pandemic; a gradual recovery of testing occurred during the post-intervention period, while Xpert MTB/RIF and DST showed a stationary trend during the intervention and post-pandemic period. It was also found that smear microscopy and HIV tests exhibited increasing trends in the post-pandemic period.

**Table 1:** Impact of COVID-19 on the temporal trend of tests performed in Brazil and its macro-regions (2010-2024).

Variable	Intervention period		Post-intervention period	
	Trend	MPC (95% CI)	Trend	MPC (95% CI)
**Brazil**				
Smear microscopy	Decreasing	–16.4 (–0.25; –0.11)	Increasing	+0.84 (0.64; 1.03)
Culture	Decreasing	–21.4 (–0.34; –0.14)	Increasing	+1.22 (1.00; 1.43)
HIV	Decreasing	–16.2 (–0.24; –0.11)	Increasing	+0.95 (0.78; 1.12)
Drug susceptibility test	Stationary	–8.9 (–0.62; 0.44)	Stationary	+0.15 (–8.97; 10.17)
Xpert MTB/RIF	Stationary	–4.3 (–1.11; 1.03)	Stationary	–0.63 (–12.34; 12.63)
**North**				
Smear microscopy	Stationary	–7.0 (–0.18; 0.03)	Increasing	+0.82 (0.39; 1.24)
Culture	Stationary	+1.6 (–0.13; 0.16)	Stationary	+0.51 (–0.12; 1.13)
HIV	Decreasing	–12.0 (–0.21; –0.04)	Increasing	+1.07 (0.74; 1.39)
Drug susceptibility test	Stationary	+9.0 (–0.45; 0.61)	Stationary	–0.87 (–8.50; 7.39)
Xpert MTB/RIF	Stationary	+11.6 (–0.77; 0.98)	Stationary	–1.16 (–9.93; 8.47)
**Northeast**				
Smear microscopy	Decreasing	–14.8 (–0.23; –0.09)	Increasing	+0.78 (0.51; 1.05)
Culture	Decreasing	–20.6 (–0.31; –0.14)	Increasing	+1.34 (1.00; 1.68)
HIV	Decreasing	–16.5 (–0.25; –0.11)	Increasing	+0.89 (0.62; 1.17)
Drug susceptibility test	Stationary	–26.8 (–1.12; 0.49)	Stationary	–1.41 (–6.14; 3.56)
Xpert MTB/RIF	Stationary	+7.0 (–0.71; 0.84)	Stationary	–1.29 (–9.81; 8.02)
**South**				
Smear microscopy	Decreasing	–18.9 (–0.27; –0.14)	Increasing	+0.73 (0.47; 0.99)
Culture	Decreasing	–26.5 (–0.37; –0.24)	Increasing	+1.17 (0.90; 1.44)
HIV	Decreasing	–19.3 (–0.27; –0.16)	Increasing	+0.88 (0.66; 1.10)
Drug susceptibility test	Stationary	+4.0 (–0.68; 0.75)	Stationary	–1.36 (–9.82; 7.90)
Xpert MTB/RIF	Stationary	+11.0 (–0.59; 0.80)	Stationary	–0.43 (–9.36; 9.38)
**Southeast**				
Smear microscopy	Decreasing	–18.3 (–0.25; –0.15)	Increasing	+0.88 (0.68; 1.08)
Culture	Decreasing	–25.9 (–0.37; –0.23)	Increasing	+1.26 (0.99; 1.53)
HIV	Decreasing	–18.1 (–0.24; –0.16)	Increasing	+0.90 (0.73; 1.07)
Drug susceptibility test	Stationary	+10.8 (-0.28; 0.48)	Stationary	–0.36 (–7.18; 6.95)
Xpert MTB/RIF	Stationary	+12.5 (-0.73; 0.97)	Stationary	–0.43 (–12.73; 13.60)
**Central-West**				
Smear microscopy	Decreasing	–16.5 (–0.32; –0.04)	Increasing	+0.56 (0.00; 1.12)
Culture	Decreasing	–16.0 (–0.32; –0.03)	Increasing	+1.56 (0.95; 2.16)
HIV	Decreasing	–15.7 (–0.27; –0.07)	Increasing	+1.17 (0.76; 1.58)
Drug susceptibility test	Stationary	–9.1 (–0.67; 0.48)	Stationary	-0.42 (–7.18; 6.83)
Xpert MTB/RIF	Stationary	+8.5 (–0.55; 0.71)	Stationary	+0.18 (–7.57; 8.58)

CI, confidence interval; MPC, monthly percent change.

### Spatial pattern shifts in diagnostic testing

**Figure 2:**
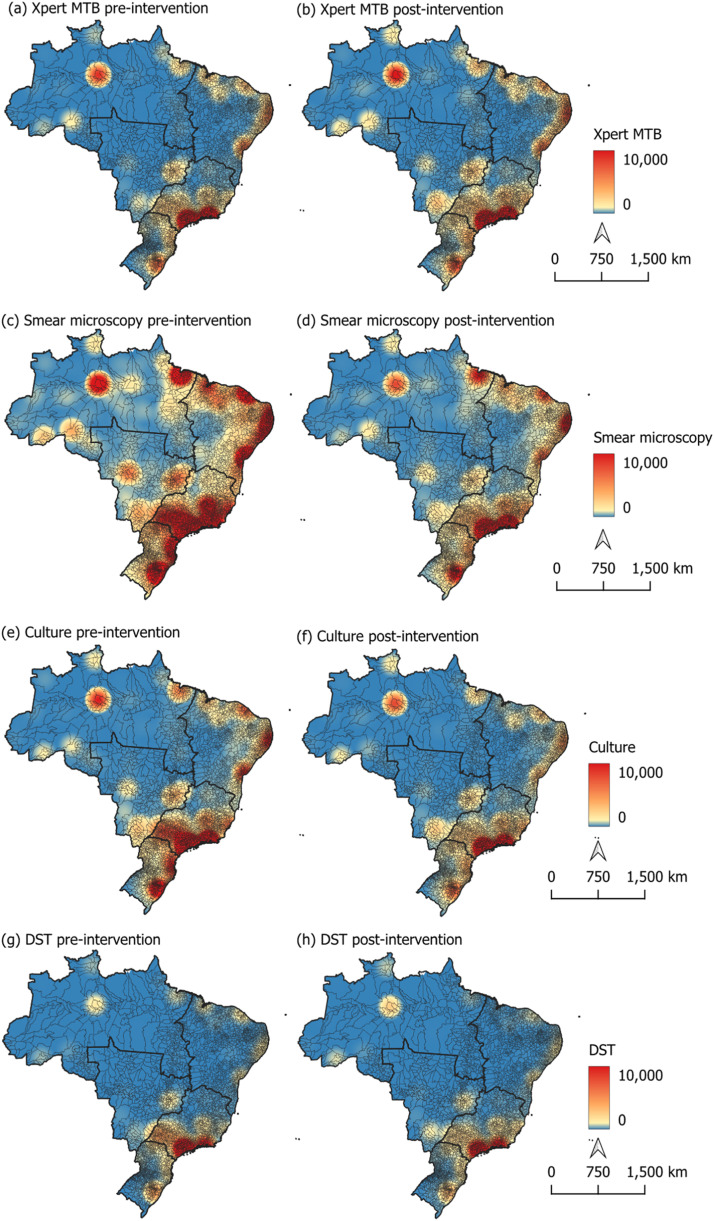
Kernel density analysis of the spatial distribution of the annual average of tuberculosis diagnostic tests during the pre-intervention period (2010-2019) and post-intervention period (2020-2024), in Brazilian municipalities.

Kernel density maps revealed marked regional disparities over time. Pre-pandemic, Xpert MTB/RIF testing was concentrated in the Southeast region, with a smaller high-density cluster in the North of the country (Figure 2a). Post-pandemic, moderate-density clusters appeared in the Central-West and in some municipalities in the Northeast (Figure 2b).

For smear microscopy (Figure 2c and 2d) and culture test (Figure 2e and 2f), the pre-intervention pattern shows high-density points in nearly all regions of the country except for the Central-West. In the post-intervention period, a sharp reduction in density is noted, revealing a similar decreasing pattern across all regions. DST (Figure 2g and 2h) showed little formation of high-density clusters, with concentrated points only in the Southeast. The other regions presented only a few municipalities with slight concentrations of tests performed.

The Global Moran’s I (Supplementary Table 2) revealed a positive and statistically significant spatial autocorrelation for the association between TB notifications and all evaluated diagnostic tests in both the pre-intervention period (2010–2019) and post-intervention period (2020–2024) (Supplementary Table 2). In the pre-intervention period, the highest autocorrelation was observed between notifications and smear microscopy (I = 0.0952; *P* = 0.002), followed by Xpert MTB/RIF (I = 0.0791; *P* = 0.002), culture (I = 0.0755; *P* = 0.001), and DST (I = 0.0659; *P* = 0.001). A similar correlation pattern was maintained in the post-intervention period.

**Figure 3:**
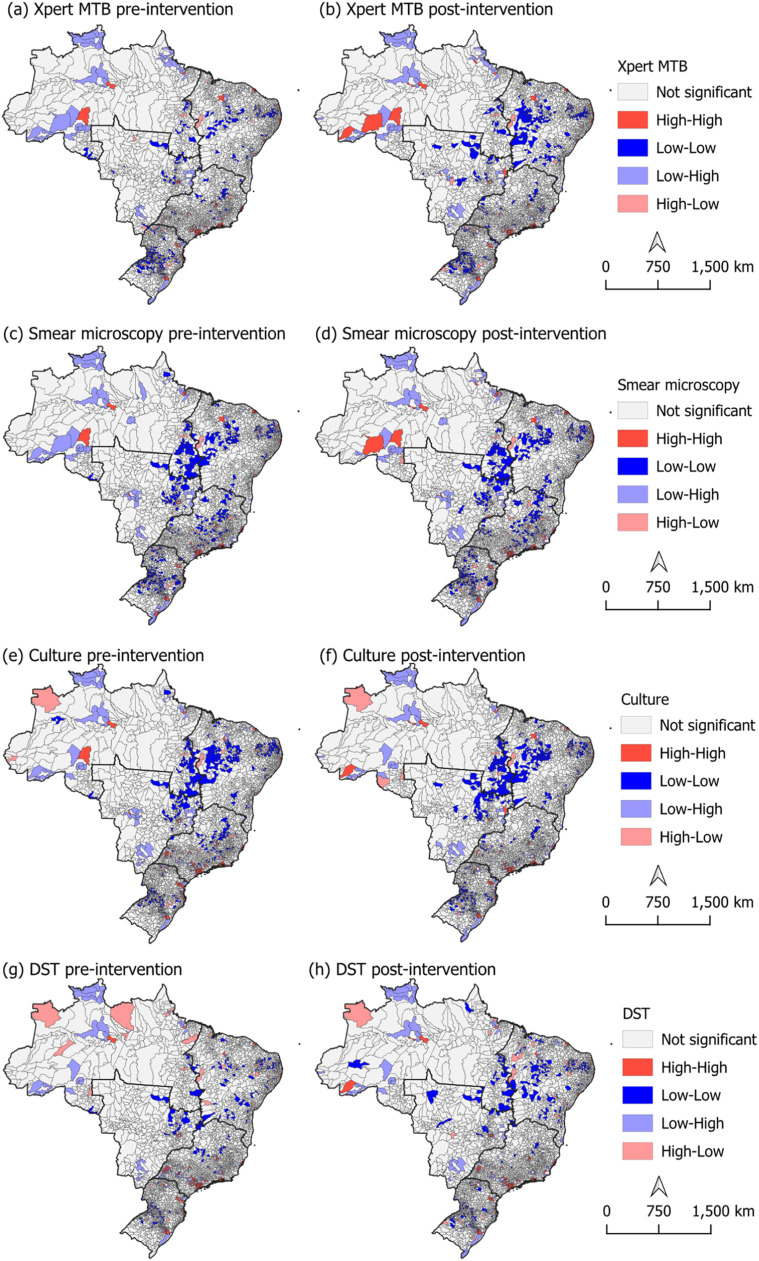
Bivariate Moran’s I analysis between tuberculosis notifications and diagnostic tests during the pre-intervention (2010-2019) and post-intervention (2020-2024) periods in Brazilian municipalities.

When analyzing the Local Moran’s I (Figure 3), the formation of municipal clusters can be seen in both the pre-intervention (2010-2019) and post-intervention (2020-2024) periods. For all tests, High-High clusters are primarily concentrated in the Southeast region, with smaller, scattered nuclei in the North. Low-Low clusters are more prevalent in parts of the Central-West and Northeast regions.

The comparison between the two periods (Figure 3) for Xpert MTB/RIF revealed autocorrelation clusters with slight spatial expansion of High-High clusters in the North, while the extent of Low-Low clusters became more evident across the country, possibly indicating an expansion in the implementation of Xpert MTB/RIF in the post-intervention period. Smear microscopy maintained a similar pattern between the two periods. Culture testing showed a slight decrease in spatial correlation in the post-intervention period, with fewer visible clusters across the territory. DST, on the other hand, showed a slight increase in Low-Low clusters in parts of the Northeast and Central-West regions.

**Figure 4:**
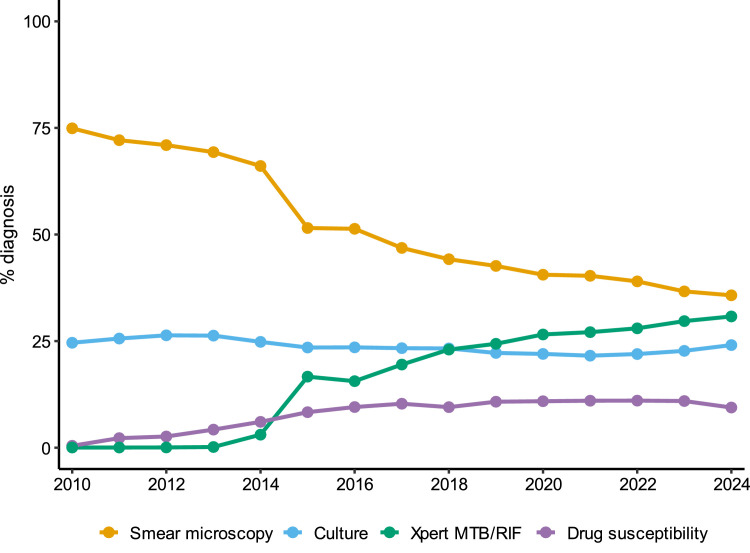
Percentage of tuberculosis diagnostic tests performed in Brazil from 2014 to 2024.

When observing the percentage of TB diagnosis over time associated with each type of test (Figure 4), a sharp decline in smear microscopy is noted in the year following the introduction of Xpert MTB/RIF in Brazil, with a peak in Xpert MTB/RIF usage observed in 2015. In the subsequent years, there is a progressive increase in the percentage of diagnoses made through Xpert MTB/RIF, accompanied by a gradual decline in smear microscopy. By 2024, both tests show similar percentages of diagnostic use, highlighting the continued growth in the use of Xpert MTB/RIF, which has progressively replaced smear microscopy.

**Figure 5:**
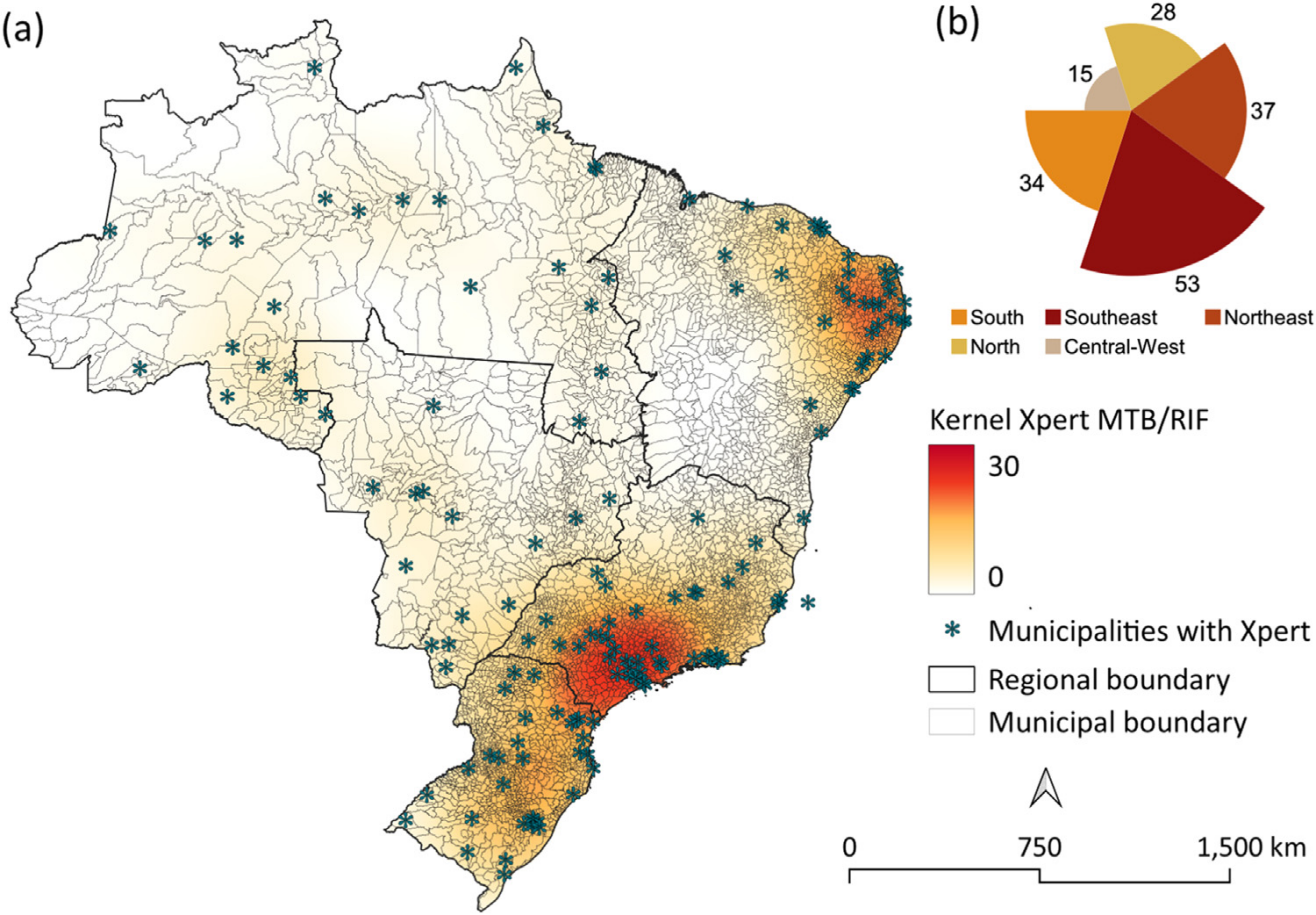
Distribution of municipalities with access to the Xpert MTB/RIF diagnostic test in Brazil during the post-intervention period.

The analysis of the distribution of laboratories equipped with Xpert MTB/RIF (Figure 5) shows that only 167 municipalities in Brazil have this equipment in their laboratory facilities, with the highest concentration in the Southeast region (n = 53; 31.7%) and Northeast (n = 37; 22.2%), followed by the South (n = 28; 16.8%), Central-West (n = 34; 20.4%), and North (n = 15; 9.0%). However, it should be taken into account that municipalities with Xpert MTB/RIF equipment provide diagnostic coverage for neighboring municipalities. Even with this expanded coverage, it is still possible to observe large areas without access – neither do they have the equipment, nor do their neighboring municipalities.

### GLS model results

GLS modeling of the impact of diagnostic tests on TB case notifications showed significant shifts from the pre- to post-pandemic (Table 2). The analysis showed that the coefficient associated with Xpert MTB/RIF increased from 0.048 ± 0.015 in the pre-intervention period (2010 to 2019) to 0.175 ± 0.031 in the post-intervention period (2020 to 2024), indicating an absolute increase of 0.127 in the impact of this test on TB diagnosis after the COVID-19 pandemic.

**Table 2:** Generalized least squares regression model for the impact of diagnostic tests on tuberculosis notification in the pre- and post-intervention periods.

Variable	Pre-intervention			Post-intervention		
	Coefficient	*t*-value	*P*-value	Coefficient	*t*-value	*P*-value
Intercept	0.234 ± 0.114	2.061	<0.041	0.643 ± 0.192	3.356	<0.001
Xpert MTB/RIF	0.048 ± 0.015	3.213	<0.002	0.175 ± 0.031	5.730	<0.001
Smear microscopy	0.928 ± 0.025	36.719	<0.001	0.693 ± 0.040	17.352	<0.001
Culture	0.075 ± 0.031	2.406	<0.017	0.208 ± 0.046	4.481	<0.001
Drug susceptibility testing	–0.036 ± 0.018	–1.947	<0.053	–0.107 ± 0.030	–3.556	<0.001

In contrast, smear microscopy decreased from 0.928 ± 0.025 to 0.693 ± 0.040, representing a drop of 0.235 in its contribution to variation in case notifications. Culture testing increased from 0.075 ± 0.031 to 0.208 ± 0.046, an increment of 0.133. Lastly, DST had a negative effect, decreasing from –0.036 ± 0.018 before the pandemic to –0.107 ± 0.030 afterward, representing a –0.071 change in the magnitude of its impact.

## Discussion

This nationwide ecological study highlights the disruption COVID-19 imposed on TB and HIV diagnostic services across Brazil from 2010 to 2024. Traditional diagnostic methods, especially smear microscopy and culture, experienced significant abrupt declines during the early pandemic phase. Molecular testing using Xpert MTB/RIF demonstrated remarkable resilience, with stationary values during the intervention and post-COVID-19 period and subsequent growth in the number of tests conducted, which resulted in a sharp reduction in the performance of the smear microscopy test.

The stability of Xpert MTB/RIF suggests greater resilience of this type of diagnostic technology in Brazil’s TB diagnostic network to the initial shock of the pandemic. This may be attributed to the operational structure of centralized laboratories and the prioritization of molecular technologies in response to the operational challenges imposed by the pandemic, given their similarity to PCR-based platforms [26]. Unlike traditional smear microscopy, Xpert MTB/RIF uses a fully automated, cartridge-based real-time PCR system, which greatly reduces the need for skilled operators and lowers biosafety risks [27]. While the Xpert MTB/RIF system requires a stable power supply and annual calibration, it delivers rapid, highly sensitive detection of TB and rifampicin resistance within approximately 2 hours, enabling much faster diagnoses [28].

The evidence of the resilience of Xpert MTB/RIF can be underscored by the adaptations laboratories had to make to confront the COVID-19 pandemic. Laboratories that previously performed 1689 reverse transcription-PCR tests in March 2020 went on to perform 7624 in May 2020, representing a 451% increase in molecular diagnostic capacity in this initial phase of the pandemic [29], marking a new phase in Brazilian laboratories defined by the expanded adoption of molecular diagnostics.

The diagnostic advantage of implementing Xpert MTB/RIF for TB detection is well documented. A retrospective study conducted at a hospital in Bahia, Brazil, demonstrated that incorporating Xpert MTB/RIF enabled the detection of 254 TB-positive cases previously classified as negative via smear microscopy. This resulted in a 59.9% increase in diagnostic gain [30]. Another study conducted in Brazil found a 63.6% increase in resistant TB case notifications and a 92.1% increase in drug-sensitive cases [31].

However, it is necessary to highlight that the increased use of molecular tests has led to a decline in smear microscopy, reflecting a significant shift in the TB diagnostic landscape driven by the broader modernization of TB diagnosis accelerated by the pandemic. This shift in the profile of the selection and application of the rapid molecular test for TB is visually illustrated in Figure 4 and quantitatively detailed in Table 2, where the use of the Xpert MTB/RIF test tripled, leading to a clear reduction in the use of sputum smear microscopy. Xpert MTB/RIF has become central to both surveillance and case confirmation; however, not all cities have implemented molecular testing for TB diagnosis, and in such cases, smear microscopy continues to be used.

The GLS regression analysis further supports this interpretation. The post-pandemic rise in the coefficient for Xpert MTB/RIF demonstrates the strengthening of molecular testing as the main driver of TB case detection in Brazil. In contrast, the decline in the contribution of smear microscopy indicates a structural shift in the diagnostic landscape. This transition reflects the broader modernization of TB diagnosis accelerated by the pandemic, as molecular methods became central to surveillance and case confirmation. These findings indicate that the health crisis may have accelerated the adoption of molecular diagnostics [32].

Despite the advances in the distribution of Xpert MTB/RIF, there is a problem associated with the implementation of Xpert MTB/RIF on a large scale, since this test is associated with high costs [33] and often depends on specific laboratory platforms and cartridge supplies [34], resulting in reliance on a limited number of centralized laboratories, highlighting inequalities in distribution and access to diagnosis via molecular testing.

The unequal distribution of Xpert MTB/RIF may indicate a geographically uneven recovery of diagnostic services after the pandemic. The pandemic may have exacerbated preexisting disparities in access to TB diagnosis, considering that the recovery process was likely influenced by the availability of molecular testing infrastructure, with vulnerable and less-resourced regions remaining underserved.

In this context, several issues associated with socio-spatial inequalities can be identified in Brazil, including political, cultural, informational, and financial factors related to the territory [35]. Considering the supply of health services and health equipment, there is a marked inequality in distribution, made especially visible during the COVID-19 pandemic. In Brazil, most resources are allocated to the South and Southeast regions, with a comparatively more homogeneous distribution across inland municipalities when contrasted with the North, Northeast, and Center-West, where resources are primarily concentrated in state capitals and consolidated urban centers [36,37].

This dependency restricts accessibility and poses challenges for large-scale implementation, particularly in resource-limited settings. Expanding molecular diagnostic options beyond exclusive platforms is critical to enhancing diagnostic capacity, enabling the detection of drug resistance and strain diversity, and promoting decentralized testing.

One way to get around this problem is to use the pooled sputum samples for molecular screening, which has emerged as an efficient approach to optimize resource utilization and reduce testing costs, mainly due to Xpert MTB/RIF [38], while maintaining high specificity, particularly among vulnerable populations, thus being able to reduce socio-spatial inequalities in the territory. It is essential to ensure a comprehensive understanding of this strategy and to provide appropriate training for personnel prior to its implementation. Therefore, such disparities underscore the need for targeted investments in underperforming regions and policies to decentralize molecular testing technologies and strengthen primary health networks to reduce those inequities [39].

Another relevant finding is the persistently negative coefficient for DST in both the pre- and post-intervention periods. This inverse association with total TB notifications likely reflects the role of DST as a secondary, confirmatory test, primarily performed among cases with suspected or confirmed drug resistance [13]. Regions with higher DST volumes may therefore correspond to areas with a greater burden of drug-resistant TB, where diagnostic efforts are directed toward treatment monitoring and resistance detection rather than new case identification.

In addition, the observed decline in DST use may be related to the gradual replacement of this conventional technique by the line probe assay, a molecular diagnostic method that has become increasingly adopted within the Brazilian laboratory network [40]. Integrating drug resistance investigation with routine diagnostic services remains essential to ensure timely case detection, optimize resource allocation, and strengthen comprehensive TB control strategies.

Furthermore, integrating drug resistance testing with routine diagnostics using advanced molecular assays can improve timely detection and treatment. To prepare for future pandemics, health systems must develop protocols that ensure uninterrupted TB diagnostic services, resource allocation that balances hospital and primary care needs, and emergency plans to adapt laboratory networks promptly [35,36].

Policy efforts should prioritize the equitable expansion of molecular diagnostic technologies across all Brazilian regions to address diagnostic access disparities [35]. This includes increasing investments in laboratory infrastructure, decentralizing molecular testing platforms to reach remote areas, and enhancing supply chain resilience to ensure continuity during emergencies, considering each reality and territorial need of the country. Moreover, strengthening the public health care network is necessary so that the implementation of new molecular technologies, such as Xpert MTB/RIF, can intensify diagnostic efforts toward the elimination of TB in the country [41]. In alignment with the *Programa Brasil Saudável* (Health Brazil Program), these actions should be embedded in a territorial equity agenda, linking financing to regional gaps, integrating laboratory networks with primary care, and adopting shared monitoring indicators [42].

This study has limitations inherent to its ecological design, which relies on aggregated municipal-level data and precludes inference at the individual level. The use of secondary data from national notification systems may be subject to reporting delays and underreporting, potentially affecting the timeliness and accuracy of observed diagnostic trends. These factors necessitate cautious interpretation of findings, particularly regarding spatial heterogeneity and temporal trend analyses, as unmeasured confounders and data completeness issues could influence the results.

The study advances knowledge by evidencing fifteen years of TB and HIV diagnostic services in Brazil, highlighting the increasing coverage of molecular tests and considerable recovery, mainly due to the expansion of Xpert MTB/RIF. However, a major challenge remains to ensure equity and universal access to molecular testing for all. Other diagnostic methods, such as microscopy, are decreasing in use, while culture and DST have remained stable.

To advance toward the End TB goals, it is important to diversify the scope of technologies by allowing the introduction of other diagnostic products and facilitating their regulation, especially point-of-care and decentralized care models within the universal health system. This must be accompanied by simultaneous efforts to address the social inequalities emphasized in the End TB Strategy and reinforced by the Health Brazil Program.

## Conclusion

This nationwide ecological study comprehensively analyzed fifteen years of TB and HIV diagnostic services in Brazil, highlighting significant disruptions caused by the COVID-19 pandemic and the uneven recovery across regions. The rapid expansion and resilience of molecular diagnostics, particularly Xpert MTB/RIF, have transformed the diagnostic landscape, replacing traditional methods such as smear microscopy. However, significant regional disparities remain, with more socioeconomically advantaged areas having better access to these advanced technologies, while vulnerable regions face persistent gaps. Advancement toward the End TB Strategy requires diversification of diagnostic technologies, streamlined regulation of innovative products, and integration of decentralized care models within Brazil’s universal health system. Furthermore, addressing the territorial inequalities emphasized in the End TB Strategy and the *Programa Brasil Saudável* (Healthy Brazil Program) will be critical for strengthening health system resilience and achieving sustained TB and HIV control throughout the country.

## Declaration of competing interest

The authors have no competing interests to declare.

## Appendix Supplementary materials

Image, application 1

Image, application 2
