# Supplementary material for: Fifteen years of tuberculosis and HIV diagnostic services in Brazil: disruption, regional disparities, and recovery before, during, and after the COVID-19 pandemic
Source: IJID Reg. 2025 Oct 31;17:100796. doi: 10.1016/j.ijregi.2025.100796 (PMC12666353; doi:10.1016/j.ijregi.2025.100796)
Supplement: Supplementary file 1 [file mmc1.docx]

**Supplementary Table 1.** Temporal trend of tests performed in Brazil and its macro-regions during the pre-COVID-19 pandemic period (2010–2019).

| **Variable** | **Prais–Winsten**  **(95%CI)** | **Trend** | **MPC**  **(95%CI)** |
| --- | --- | --- | --- |
| **Brazil** | | | |
| Smear microscopy | <0.01  (0.00; 0.01) | Decreasing | -0.06  (-0.10; -0.01) |
| Culture | <0.01  (0.00; 0.01) | Increasing | +0.33  (0.29; 0.38) |
| HIV | <0.01  (0.00; 0.01) | Increasing | +0.27  (0.23; 0.30) |
| Drug susceptibility test | 0.10  (0.01; 0.06) | Stationary | +2.39  (-0.43; 5.28) |
| Xpert MTB | <0.05  (0.02; 0.06) | Increasing | +4.77  (1.98; 7.63) |
| **North** | | | |
| Smear microscopy | <0.01  (0.00; 0.01) | Increasing | +0.11  (0.05; 0.18) |
| Culture | <0.01  (0.00; 0.01) | Increasing | +0.58  (0.51; 0.65) |
| HIV | <0.01  (0.00; 0.01) | Increasing | +0.53  (0.48; 0.58) |
| Drug susceptibility test | <0.05  (0.01; 0.06) | Increasing | +3.21  (0.73; 5.75) |
| Xpert MTB | <0.05  (0.02; 0.06) | Increasing | +4.27  (2.15; 6.44) |
| **Northeast** | | | |
| Smear microscopy | <0.01  (0.00; 0.01) | Decreasing | -0.10  (-0.15; -0.04) |
| Culture | <0.01  (0.00; 0.01) | Increasing | +0.20  (0.13; 0.27) |
| HIV | <0.01  (0.00; 0.01) | Increasing | +0.30  (0.26; 0.34) |
| Drug susceptibility test | <0.05  (0.02; 0.04) | Increasing | +3.04  (1.74; 4.36) |
| Xpert MTB | <0.05  (0.02; 0.07) | Increasing | +4.40  (1.83; 7.04) |
| **South** | | | |
| Smear microscopy | <0.01  (0.00; 0.01) | Decreasing | -0.08  (-0.13; -0.03) |
| Culture | <0.01  (0.00; 0.01) | Increasing | +0.31  (0.25; 0.36) |
| HIV | <0.01  (0.00; 0.01) | Increasing | +0.12  (0.07; 0.16) |
| Drug susceptibility test | <0.05  (0.01; 0.05) | Increasing | +3.01  (0.87; 5.20) |
| Xpert MTB | <0.05  (0.02; 0.06) | Increasing | +4.17  (1.75; 6.65) |
| **Southeast** | | | |
| Smear microscopy | <0.01  (0.00; 0.01) | Decreasing | -0.06  (-0.11; -0.02) |
| Culture | <0.01  (0.00; 0.01) | Increasing | +0.33  (0.27; 0.39) |
| HIV | <0.01  (0.00; 0.01) | Increasing | +0.23  (0.20; 0.26) |
| Drug susceptibility test | 0.15  (-0.01; 0.05) | Stationary | +2.19  (-0.73; 5.19) |
| Xpert MTB | <0.05  (0.02; 0.07) | Increasing | +4.32  (1.20; 7.54) |
| **Central-West** | | | |
| Smear microscopy | <0.01  (0.00; 0.01) | Decreasing | -0.09  (-0.17; -0.01) |
| Culture | <0.01  (0.00; 0.01) | Increasing | +0.46  (0.36; 0.56) |
| HIV | <0.01  (0.00; 0.01) | Increasing | +0.35  (0.29; 0.41) |
| Drug susceptibility test | <0.05  (0.01; 0.05) | Increasing | +2.91  (1.12; 4.73) |
| Xpert MTB | <0.05  (0.02; 0.05) | Increasing | +3.50  (1.78; 524) |

Legend: 95% CI = 95% confidence interval; MPC = monthly percent change.
