# Supplementary material for: Fifteen years of tuberculosis and HIV diagnostic services in Brazil: disruption, regional disparities, and recovery before, during, and after the COVID-19 pandemic
Source: IJID Reg. 2025 Oct 31;17:100796. doi: 10.1016/j.ijregi.2025.100796 (PMC12666353; doi:10.1016/j.ijregi.2025.100796)
Supplement: Supplementary file 2 [file mmc2.docx]

**Supplementary Table 2.** Spatial autocorrelation analysis between tuberculosis notifications and diagnostic tests in the pre-intervention period (2010–2019) and post-intervention period (2020–2024)

| Variables | Moran’s I | Mean | Standard Deviation | p-value | z-value |
| --- | --- | --- | --- | --- | --- |
| Pre-intervention | | | | | |
| TB x Xpert MTB/RIF | 0.0791 | 0.0001 | 0.0060 | 0.002 | 13.1405 |
| TB x Smear microscopy | 0.0952 | 0.0001 | 0.0067 | 0.002 | 14.1125 |
| TB x Culture | 0.0755 | 0.0002 | 0.0060 | 0.001 | 12.6772 |
| TB x DST | 0.0659 | 0.0001 | 0.0059 | 0.001 | 11.1729 |
| Post-intervention | | | | | |
| TB x Xpert MTB/RIF | 0.0788 | 0.0001 | 0.0061 | 0.001 | 12.9406 |
| TB x Smear microscopy | 0.0962 | 0.0001 | 0.0067 | 0.002 | 14.4287 |
| TB x Culture | 0.0731 | 0.0001 | 0.0060 | 0.001 | 12.1718 |
| TB x DST | 0.0613 | 0.0001 | 0.0057 | 0.001 | 10.8593 |
